# *De novo* Analysis of the Epiphytic Transcriptome of the Cucurbit Powdery Mildew Fungus *Podosphaera xanthii *and Identification of Candidate Secreted Effector Proteins

**DOI:** 10.1371/journal.pone.0163379

**Published:** 2016-10-06

**Authors:** David Vela-Corcía, Rocío Bautista, Antonio de Vicente, Pietro D. Spanu, Alejandro Pérez-García

**Affiliations:** 1 Instituto de Hortofruticultura Subtropical y Mediterránea “La Mayora”, Universidad de Málaga, Consejo Superior de Investigaciones Científicas (IHSM−UMA−CSIC), Departamento de Microbiología, Facultad de Ciencias, Málaga, Spain; 2 Plataforma Andaluza de Bioinformática, Edificio de Bioinnovación, Parque Tecnológico de Andalucía, Málaga, Spain; 3 Department of Life Sciences, Imperial College London, United Kingdom; Agriculture and Agri-Food Canada, CANADA

## Abstract

The cucurbit powdery mildew fungus *Podosphaera xanthii* is a major limiting factor for cucurbit production worldwide. Despite the fungus’s agronomic and economic importance, very little is known about fundamental aspects of *P*. *xanthii* biology, such as obligate biotrophy or pathogenesis. To design more durable control strategies, genomic information about *P*. *xanthii* is needed. Powdery mildews are fungal pathogens with large genomes compared with those of other fungi, which contain vast amounts of repetitive DNA sequences, much of which is composed of retrotransposons. To reduce genome complexity, in this work we aimed to obtain and analyse the epiphytic transcriptome of *P*. *xanthii* as a starting point for genomic research. Total RNA was isolated from epiphytic fungal material, and the corresponding cDNA library was sequenced using a 454 GS FLX platform. Over 676,562 reads were obtained and assembled into 37,241 contigs. Annotation data identified 8,798 putative genes with different orthologues. As described for other powdery mildew fungi, a similar set of missing core ascomycete genes was found, which may explain obligate biotrophy. To gain insight into the plant-pathogen relationships, special attention was focused on the analysis of the secretome. After this analysis, 137 putative secreted proteins were identified, including 53 candidate secreted effector proteins (CSEPs). Consistent with a putative role in pathogenesis, the expression profile observed for some of these CSEPs showed expression maxima at the beginning of the infection process at 24 h after inoculation, when the primary appressoria are mostly formed. Our data mark the onset of genomics research into this very important pathogen of cucurbits and shed some light on the intimate relationship between this pathogen and its host plant.

## Introduction

Powdery mildew fungi (*Erysiphales*) are plant pathogens that cause powdery mildew diseases in many plant species, such as cereals, fruits and vegetable crops as well as ornamentals [1]. These fungi are ascomycetes and obligate biotrophs; that is, their growth and reproduction depend on their living hosts. As a consequence, the fungi cannot be cultured *in vitro*, posing serious challenges for experimentation [2]. Recently, the genomes of five powdery mildew species were sequenced [3–5]. Analyses showed that the genomes bear hallmarks of the obligate biotrophic lifestyle. Essentially, the genomes are very large (>120 Mb) and full of retrotransposons, potentially allowing for high genomic flexibility. Notably, the genomes lack a considerable number of genes otherwise present in ascomycetes, which may explain why powdery mildew fungi rely on living host plants for propagation. The powdery mildew genomes encode a significant number of species-specific candidate secreted effector proteins (CSEPs), which may represent the weapons of powdery mildews for pathogenesis [3]. In addition, the fast adaptation of powdery mildews to their host species appears to be based on a diverse haplotype pool that provides great genetic potential for pathogen variation [4]. Moreover, in powdery mildew genomes, copy number variations can be adaptive in the development of resistance to fungicides by providing increasing quantitative protection in a gene-dosage-dependent manner [5].

The obligate and intimate relationships of powdery mildews with their plant hosts impose a strong selection pressure on these parasites to develop strategies to successfully infect while evading host detection and defence mechanisms. The molecules in charge of this process are called effectors. Effectors are proteins that enhance disease development by targeting host processes but are otherwise redundant to basal growth processes in the pathogen [6]. Secreted protein effectors influence host metabolism, defence mechanisms and modify the host cell structure to provide an environment for successful infection [7,8]. Effectors are generally small proteins in their mature form, and they rarely have homologues in more remotely related microbial species [9].

Effectors are broadly divided into apoplastic and cytoplasmic depending on their final destination in the host. Apoplastic effectors often exhibit inhibitory activity against extracellular host hydrolytic enzymes (e.g., proteases) and are typically small and highly cysteine-rich secreted proteins. Most cytoplasmic effector proteins have been identified through their avirulence functions, i.e., based on their genotype-specific recognition by matching plant resistance proteins [10]. In one class of oomycete effectors, an amino acid motif (RxLR-EER) is required as a translocation signal for delivery of effector proteins from haustoria into host plant cells. This motif is located a few amino acids downstream of the signal peptide cleavage sites [11]. Similarly, an amino acid motif, Y/F/WxC, has been found in the N-terminal, downstream of signal peptide cleavage site, in hundreds of powdery mildew effector candidates [6,10]; the biological significance of this motif is currently unknown.

The cucurbit pathogen *Podosphaera xanthii* is considered the main causal agent of powdery mildew on cucurbits worldwide and one of the most important limiting factors for cucurbit production in Spain [12,13]. Most research has focused on different aspects of disease control; unfortunately, however, powdery mildew continues to impose serious limitations on cucurbit production throughout the world. Despite the agronomic and economic importance of the fungus, very little is known about the physiological and molecular processes involved in *P*. *xanthii* biology and pathogenesis [14]. To design novel and more durable control strategies, genomic information of *P*. *xanthii* is needed. Until very recently, powdery mildew genomics remained elusive to researchers. Fortunately, the advent of new “omic technologies” is alleviating this situation.

The use of expressed sequence tags (ESTs) derived from protein-coding mRNA sequences is a useful approach for gene discovery; however, this method is obsolete compared with the next-generation sequencing (NGS) platforms because the throughput of NGS provides a massive amount of information. One of the most important challenges in NGS is *ab initio* construction of the transcriptome of an organism for which the genome sequence is not available. Thus, transcriptome studies help gene discovery and provide novel insight into various unique species-specific, biological processes/pathways [15]. Although the genome of *P*. *xanthii* is not available, the present study provides insight into the *P*. *xanthii* transcriptome. We describe the results of the 454 sequencing of a pooled RNA sample obtained from mycelia and conidia of *P*. *xanthii* infecting zucchini cotyledons and *de novo* assembly and annotation of the transcriptome data. In addition, the data were analysed to identify missing genes (compared with baker’s yeast) in powdery mildew fungi and the pool of secreted proteins (including candidate effectors), which are possibly involved in pathogenesis.

## Materials and Methods

### Plant material and fungal isolate

Zucchini plants (*Cucurbita pepo*) from cv. Negro Belleza (Semillas Fitó, Barcelona, Spain), a cultivar highly susceptible to powdery mildew, were grown from seeds in a growth chamber at 25°C over a 16–8 h photoperiod. The *P*. *xanthii* isolate 2086 was propagated on zucchini cotyledons previously disinfected and maintained *in vitro* as previously described [16]. The isolate was stored at -80°C until use [17].

### RNA extraction, library synthesis and sequencing

Total RNA was isolated from epiphytic mycelium and conidia of *P*. *xanthii* collected from two different heavily powdery mildew-infected zucchini cotyledons by carefully removing the epiphytic fungal biomass with a spatula, immediately frozen in liquid nitrogen, and stored at -80°C until use. Total RNA was extracted using TRI Reagent (Sigma-Aldrich, Saint Louis, MO) and NucleoSpin RNA Plant (Macherey-Nagel, Düren, Germany) according to the manufacturer´s instructions. Total RNA was quantified using a NanoDrop 2000 spectrophotometer (Thermo Fisher Scientific, Waltham, MA). RNA quality and quantity were measured by running 1 μl of sample on an Agilent Bioanalyzer 2100 using a RNA Pico 600 chip (Agilent Technologies, Santa Barbara, CA). A non-normalised cDNA library was synthesised from 1.5 μg total RNA with the Mint-2 cDNA synthesis kit (Evrogen, Moscow, Russia). The 454 libraries obtained in this manner were immobilised on beads and clonally amplified using the GS FLX Titanium LV emPCR kit (454 Life Sciences, Branford, CT). The libraries were then sequenced using the GS FLX Titanium Sequencing Kit XLR70 (454 Life Sciences) and GS FLX Titanium PicoTiterPlate kit on a GS FLX instrument (454 Life Sciences). All kits were used according to the manufacturer’s instructions.

### *De novo* assembly, annotation and ontology

Before assembly, 454 reads were pre-processed by SeqTrimNext v.2.0.54 pipeline (http://www.scbi.uma.es/seqtrimnext), available at the Plataforma Andaluza de Bioinformática (University of Málaga, Spain), using plugins to remove low and high size sequences, adapters, poly-A, ribosomes and contaminants from plants and bacteria, as well as sequences with low quality, ambiguity and low complexity. The detailed workflow for transcriptome pre-processing and assembly is depicted in S1 Fig.

Two different programs were used to assemble the filtered sequences, MIRA3 [18] and Euler [19]. Briefly, 676,562 sequences were assembled by the MIRA3 assembler version 3 (job= “denovo,est,normal,454”, -CL: ascdc, 454_SETTINGS, -CO:fnicpst = yes, -notraceinfo, COMMON_SETTINGS, -GE: not). The same number of initial reads was assembled by Euler; however, this program uses *de Bruijn* graphs to build contigs (k-mer = 29). Contigs resulting from Eulerian assembly were mapped against cleaned reads using Bowtie 2 [20], resulting in two groups of contigs: mapped and unmapped. Both unmapped and debris contigs were submitted to FulLengtherNext (http://www.scbi.uma.es/fulllengthernext) available at the Plataforma Andaluza de Bioinformática (-g: fungi -q -c: 100 -w: 32 -s: 10.243), to identify coding sequences susceptible to rescue. Thus, contigs from MIRA3 and Euler mapped, unmapped-coding, MIRA3 debris-coding and MIRA3-coding contigs were merged by CAP3 [21] (-p-value: 95 -overlapping: 100), and individual sequences reads that had no significant overlap with any other read were classified as “singletons”. Three different annotation programs were used to annotate the pool of merged contigs renamed as unigenes, FulLengtherNext, Sma3s [22] and AutoFACT [23]. These annotators were used to annotate predicted genes using default parameters. GO terms were retrieved from the annotation process and used for the *de novo* functional annotation of predicted proteins using a GO terms mapper (http://go.princeton.edu). For visualisation, sequences were grouped into categories according to the GOSlim file for *Candida albicans* (http://go.princeton.edu/cgi-bin/GOTermMapper).

In addition, the most expressed genes and biological processes in the *P*. *xanthii* epiphytic cDNA library were also analysed. To identify the most expressed genes, trimmed reads were mapped against the contigs obtained after the assembly process using Bowtie 2. To identify the most expressed biological processes, the number of average reads per gene within the different GO categories was calculated.

### Secretome identification

Putative secreted proteins were predicted from coding contigs, both with and without orthologue, following the workflow illustrated in S2 Fig [24]. Briefly, all putative proteins with a SignalP D-score = Y (SignalP v4.1; http://www.cbs.dtu.dk/services/SignalP/) and a TargetP Loc = S (TargetP v1.1; http://www.cbs.dtu.dk/services/TargetP/) were combined. These proteins were then scanned for transmembrane spanning regions using TMHMM (TMHMM v2.0; http://www.cbs.dtu.dk/services/TMHMM/), and all proteins with 0 transmembrane domain (TM) or 1 TM, if located in the predicted N-terminal signal peptide, were kept. GPI-anchored proteins were predicted by big-PI (http://mendel.imp.ac.at/gpi/gpi_server.html). All proteins predicted as extracellular were retained in the final secretome dataset. WolfPSort analysis (Ext >17) was performed using ‘‘runWolfPsortSummary fungi” in the WolfPSort v0.2 package. All of those proteins were arranged by their putative biological function according to the GO terms assigned in the annotation process mentioned previously. Pfam analysis was performed using the Pfam database (http://pfam.sanger.ac.uk/search). Putative secreted proteins with an identity percentage less than 45%, after Pfam analysis, were considered to have no functional annotation. These *P*. *xanthii* proteins lacking any conserved domain and any assigned functional annotation were named as candidate secreted effector proteins (CSEPs).

### Phylogenetic and natural selection analyses of effector candidates

The CSEPs and some *P*. *xanthii* secreted proteins previously identified in other fungal pathogens to have a role in pathogenesis were aligned using the slow and accurate pairwise alignment in CLUSTALW2 (www.ebi.ac.uk/Tools/msa/clustalw2/). The alignment file was used for phylogenetic analysis *via* the phylogeny option of CLUSTALW2. The neighbour-joining algorithm [25] was chosen to generate a tree file that was subsequently fed into MEGA5 [26] for visualisation. A bootstrap consensus tree was inferred from 1000 replicates [27]. After alignment, the amino acid sequences of CSEPs were also screened manually to recognise the conserved N-terminal motif Y/F/WxC located after the signal peptide described previously in many candidate effector proteins in powdery mildew fungi [10].

### Effector candidate gene expression by quantitative RT-PCR

One-week-old zucchini cotyledons were inoculated with *P*. *xanthii* as described previously [16]. Briefly, inoculation of *P*. *xanthii* conidia was performed by depositing four conidia in each cotyledon with a sterile eyelash using a binocular microscope. Infected leaf material was harvested at different time points, frozen in liquid nitrogen and ground with a mortar and pestle. Total RNA was extracted from 100 mg tissue as mentioned previously. cDNA was synthesised using Invitrogen Superscript III Reverse Transcriptase (Thermo Fisher Scientific) with random primers according to the manufacturer’s instructions. qRT-PCR reactions were carried out with three technical replicates of cDNA corresponding to 75 ng RNA samples in an iQ5Cycler (Bio-Rad, Hercules, CA). The iQSybr Mastermix (Bio-Rad) was used with a final concentration of 0.5 μM for each primer (S1 Table), and amplification was conducted according to the following protocol: denaturation at 95°C for 3 min, then 40 cycles of 95°C for 20 s, 60°C for 20 s and 72°C for 10 s. Subsequently, melting curve analysis of between 72 and 95°C in 0.5°C steps with a dwell time of 10 s per step was performed to verify the amplification of single amplicons. In addition, amplicons were visualised on 2% agarose gels. Expression was calculated relative to the β–tubulin gene *PxTUB*2 [28] using the ΔΔCt method [29].

## Results

### Construction, sequencing and annotation of an epiphytic cDNA library of *P*. *xanthii*

Epiphytic mycelia and conidia of *P*. *xanthii* were removed from heavily infected zucchini cotyledons after 7 dpi. Total RNA was extracted, and a non-normalised cDNA library constructed. The library was sequenced by a 454 GS-FLX Titanium platform, yielding 975,070 sequence reads. To filter and discard any contaminant sequences such as vectors and adaptors used for sequencing and contaminants sequences from the host plant, these raw data were processed by SeqTrimNext v.2.0.54. After removing all contaminant sequences and quality filtering, 676,562 reads were used for the final assembly into 36,506 contigs by the MIRA3 assembler and into 11,129 contigs by the Euler assembler. In addition, coding contigs were retrieved from unmapped contigs identifying potential ORFs by FulLengtherNext, specifically, 12,193 contigs from MIRA3 and 87 from unmapped contigs. All these sequences were assembled, merged by CAP3 and submitted to NCBI screening and manual curation, yielding a total of 37,241 contigs (including singletons), henceforth named unigenes.

Derived hypothetical proteins were annotated by FulLengtherNext, Sma3s and AutoFACT simultaneously (Table 1). Sma3s provided gene ontology (GO) terms. In the case of FulLengtherNext, 10,507 proteins (28.3%) had detectable orthologues, of which 6,645 had orthologues with different IDs, and 26,655 proteins (71.7%) had no detectable orthologues. Within the group of unigenes with orthologues, there are different unigenes with the same orthologue, those unigenes are considered as paralogs due to duplication events within the *P*. *xanthii* genome. However, in order to find those unigenes that are unique, without repetitions, we used the program FulLengtherNext that pulls out all those unigenes, which is considered as the representative transcriptome.

**Table 1:** Summary statistics of the annotation process of the *P*. *xanthii* epiphytic transcriptome.

Annotation program	With orthologue		Different orthologue IDs		
	N ^a^	% ^b^	N	%	%
FulLengtherNext	10,507	28.3	6,645	63.2	71.7
Sma3s	12,918	34.8	-^c^	-	65.2
AutoFACT	15,583	57.6	- ^c^	-	42.4

^a^ Number of putative unigenes.

^b^ Percentage of annotated unigenes.

^c^ These annotation programs cannot provide those data.

When AutoFACT was used, 9,126 proteins (23.2%) lacked any detectable homologues in the NCBInr database; the remaining proteins, 12,546 (31.9%) had homologues in other species but no functional annotation, and 17,671 (44.9%) could be functionally annotated. Finally, Sma3s was used to assign all of the GO terms associated with related proteins. Among the 37,241 unigenes, 12,918 (34.8%) displayed at least one blast hit and the remaining 65.2% (24,244 unigenes) had no blast hits. From the sequences showing protein matches, 10,506 (76%) had at least one GO assigned term and 3,323 (24%) had a blast hit but no GO term assigned.

This Transcriptome Shotgun Assembly project has been deposited at DDBJ/EMBL/GenBank under the accession GEUO00000000. The version described in this paper is the first version, GEUO01000000.

### Functional classification of annotated unigenes

**Fig 1:**
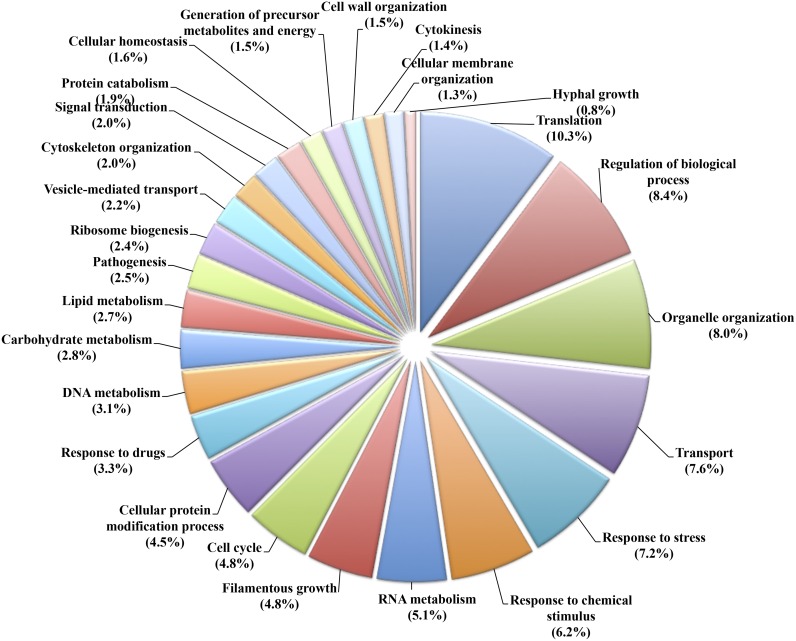
Pie chart representing the functional annotation of predicted proteins from the epiphytic transcriptome of *P*. *xanthii*. The diagram displays the relative abundance of *C*. *albicans* GOSlim categories among the 6,645 annotated predicted proteins. All data were submitted to the NCBI database under the accession GEUO00000000.

All predicted proteins with a biological process assignment were grouped into categories using the GOSlim terms generated from the *Candida albicans* genome (Fig 1). Most of annotated sequences represented elements associated with translation, regulation of biological processes, organelle organization and transport (34.3% of the library); however, responses to stress and chemical stimulus also appeared to be highly represented (13.4%). Transcripts encoding proteins involved in primary metabolism (RNA, DNA, carbohydrate and lipid metabolism as well as protein catabolism and generation of precursor metabolites and energy) represented 17.1% of the library. Smaller subsets of predicted proteins were involved in signal transduction, vesicle-mediated transport and other functions such as pathogenesis or response to drugs.

**Fig 2:**
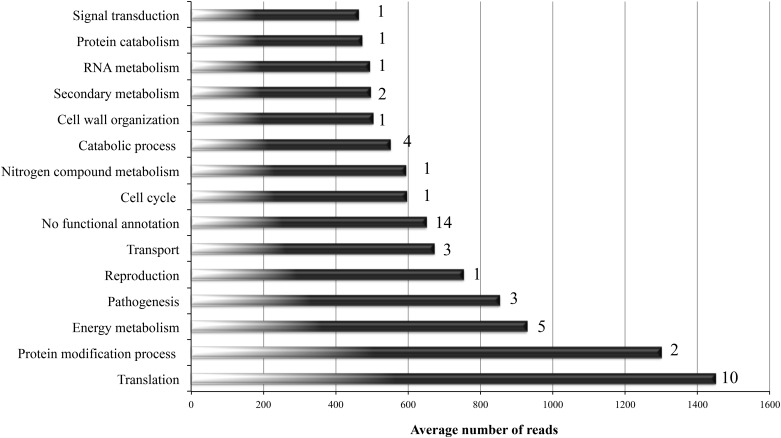
The most expressed GO categories in the epiphytic transcriptome of *P*. *xanthii*. Representation of the number of average reads within the most expressed GO categories in the *P*. *xanthii* epiphytic cDNA library. For each category, the number of genes within the group of Top50 most expressed transcripts is shown on the right side of the bars. See S2 Table for details on Top50 most expressed genes.

Besides functional classification, the library was analysed to identify the most expressed genes and biological process. Most expressed biological processes are presented in Fig 2. As shown, processes such as translation and protein modification were the most overrepresented with more than 1,200 average reads per gene. Besides these processes, GO categories such as energy metabolism, cell wall organisation, pathogenesis and catabolic process were also highly expressed, showing average reads of more than 800 reads per gene. The Top50 most expressed genes are shown in S2 Table. According to previously mentioned, the GO category translation contains ten of the top50 most expressed transcripts in the library, including the most expressed one, the corresponding to elongation factor 1-alpha with 2,591 reads.

### Metabolic pathways missing in *Podosphaera xanthii*

As previously described, there are missing metabolic pathways in powdery mildews.

The missing genes encode enzymes of primary and secondary metabolism, carbohydrate-active enzymes, and transporters, probably reflecting their redundancy in an exclusively biotrophic life-style [3]. Therefore, in order to evaluate the similarity of the *P*. *xanthii* sequence data to previously released powdery mildew genomic and transcriptomic datasets, a search for genes absent in mildews but present in other ascomycetes such as *Saccharomyces cerevisiae* and phytopathogens such as *Colletotrichum higginsianum*, *Magnaporthe oryzae*, and *Sclerotinia sclerotiorum*, was performed using the list of missing metabolic pathways in powdery mildew fungi raised in a previous study [3]. The authors proposed a list of 99 yeast genes that were missing in mildews; the set of metabolic pathways represented by these genes were named MACGs (missing ascomycete core genes). To identify *P*. *xanthii* MACG genes, the transcriptome of *P*. *xanthii* was compared with the *Blumeria graminis* annotated coding sequences dataset (www.blugen.org) and the *S*. *cerevisiae* genome (S3 Table). The MACGs represent a diverse set of metabolic and regulatory proteins affecting multiple processes and pathways, such as anaerobic fermentation, biosynthesis of glycerol from glycolytic intermediates, and inorganic nitrogen (nitrate) assimilation.

Although the majority of MACGs in *P*. *xanthii* coincide with those identified elsewhere, there are notable exceptions: Three predicted proteins are present in *S*. *cerevisiae* as well as in *P*. *xanthii* but not in *B*. *graminis*. These proteins are: THI4 (55% identity), which encodes a thiazole synthase and catalyses the formation of a thiazole intermediate during thiamine biosynthesis; PPS1 (45% identity), which encodes a protein phosphatase with specificity for serine, threonine, and tyrosine residues and plays a role in the DNA synthesis phase of the cell cycle; and FDH1 (70% identity), a protein that encodes a NAD-dependent formate dehydrogenase, which may protect cells from exogenous formate.

### Characterisation of the *P*. *xanthii* epiphytic secretome

**Fig 3:**
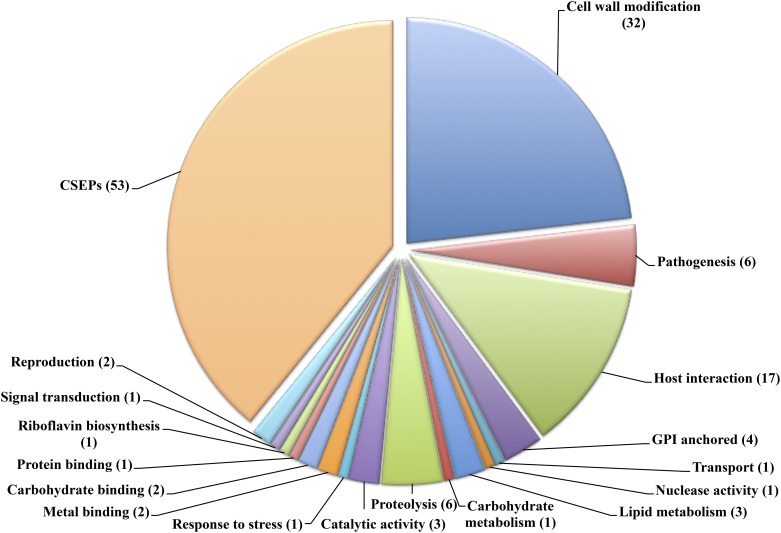
Pie chart representing the functional annotation of the *P*. *xanthii* secretome deduced from the predicted proteins from the epiphytic transcriptome. The diagram displays the relative abundance of GO categories among the total of 137 putatively secreted proteins identified; 84 secreted proteins were grouped by biological functions, and 53 had no GO terms and, consequently, were catalogued as candidate secreted effector proteins (CSEPs). The number of proteins within each functional category is presented in brackets. See Table 2 and S4 Table for details.

Five different programs were used to identify the secretome of *P*. *xanthii* (S2 Fig). A total of 137 putative secreted proteins were identified. Those putative proteins were arranged by their biological function according to the GO terms assigned in the annotation process (Fig 3). Among these proteins, 84 putative secreted proteins are similar to proteins in the publicly accessible database (Table 2 and S4 Table). The deduced proteins (53) to which no GO term was assigned were then considered effector candidates. Most of them lack any conserved domain after inspection with Pfam and BlastP and, therefore, they were named as candidate secreted effector proteins (CSEPs) (Table 3).

**Table 2:** Annotation of the epiphytic secretome of *P*. *xanthii*. Only categories “Pathogenesis” and “Host interaction” are presented. Rest of categories is shown in S4 Table.

Sequence ID	Description	E-value^a^	Sequence identity (%)	Subject ID^b^	Protein length (aa)
**Pathogenesis**					
Contig2835	Cerato-platanin domain containing protein	1.00E-40	53% (63/117)	O74238	136
Contig8430	CFEM domain protein. *Blumeria graminis* f. sp. *hordei*	6.00E-34	48%(78/162)	AEQ16462	214
Contig3096	CFEM domain protein. *Fusarium verticillioides*	4.00E-22	47% (56/119)	EWG37445	1027
Contig2707	CFEM multi-domain protein. *Golovinomyces orontii*	1.00E-11	37% (26/70)	G9BES2	121
Contig7324	CAP domain. *Colletotrichum graminicola*	6.00E-32	46% (77/164)	E3QP32	438
Contig1920	glyoxal oxidase *Blumeria graminis* f. sp. *hordei* DH14	0.00E+00	77% (582/754)	CCU82032	1186
**Host Interaction**					
7790_euler	GEgh16 protein DUF domain 3129	2.00E-85	80% (122/152)	Q00638	229
Contig2158	GEgh16 protein DUF domain 3129	6.00E-97	82% (164/199)	Q00638	224
Contig5191	GEgh16 protein DUF domain 3129	3.00E-85	81% (95/116)	Q00638	287
Contig7025	GEgh16 protein DUF domain 3129	5.00E-97	82% (164/198)	Q00638	224
Contig9129	GEgh16 protein DUF domain 3129	4.00E-95	80% (161/199)	Q00638	228
Contig9350	GEgh16 protein DUF domain 3129	6.00E-97	82% (164/199)	Q00638	224
Contig_c12296_mira	GEgh16 protein DUF domain 3129	1.00E-89	79% (122/153)	Q00638	242
Contig_c1666_mira	GEgh16 protein DUF domain 3129	9.00E-97	82% (164/198)	Q00638	228
Contig_c17975_mira	GEgh16 protein DUF domain 3129	2.00E-88	79% (121/153)	Q00638	246
Contig_c18264_mira	GEgh16 protein DUF domain 3129	2.00E-91	80% (123/153)	Q00638	243
Contig_c1961_mira	GEgh16 protein DUF domain 3129	2.00E-95	81% (162/199)	Q00638	228
Contig_c3514_mira	GEgh16 protein DUF domain 3129	6.00E-97	82% (164/198)	Q00638	238
Contig_c4204_mira	GEgh16 protein DUF domain 3129	3.00E-86	83% (97/116)	Q00638	283
Contig_c8248_mira	GEgh16 protein DUF domain 3129	5.00E-97	82% (164/199)	Q00638	218
Contig5469	Putative Egh16H1 isoform DUF domain 3129	7.00E-97	61% (172/278)	Q9C1F7	303
Contig2112	Putative Egh16H1 isoform B	7.00E-49	77%(65/84)	Q9C1F6	118
Contig_c33477_mira	Putative Egh16H1 isoform B	5.00E-92	74%(145/194)	Q9C1F6	230

^a^ E-values were obtained after Blast analysis

^b^ GenBank accession number

**Table 3:** List of CSEPs identified in the *P*. *xanthii* epiphytic secretome and orthologues present in the genome of *B*. *graminis* f. sp. *hordei*.

*P*. *xanthii* CSEPs	Sequence ID	Protein length (aa)	Sequence identity (%)	*Bgh* CSEP ID
CSEP001	Contig1176	149		
CSEP002	Contig1615	286	64.19	bgh00340
CSEP003	Contig1857	271		
CSEP004	Contig1881	322	64.19	bgh00340
CSEP005	Contig1989	332		
CSEP006	Contig2616	202		
CSEP007	Contig2718	60		
CSEP008	Contig2838	296	56.67	bgh05787
CSEP009	Contig2978	130		
CSEP010	Contig3019	188	67.94	bgh04121
CSEP011	Contig3349	333	74.37	bgh01390
CSEP012	Contig3540	558	74.37	bgh01390
CSEP013	Contig3846	179	45.73	bghG002404000001001
CSEP014	Contig4458	282	64.76	bgh03043
CSEP015	Contig5094	335	49.49	bgh01899
CSEP016	Contig5882	250		
CSEP017	Contig6477	202		
CSEP018	Contig7266	218	66.21	bgh02065
CSEP019	Contig7690	194		
CSEP020	Contig7880	202		
CSEP021	Contig8160	218		
CSEP022	Contig9180	95		
CSEP023	Contig9299	202		
CSEP024	Contig_c3638_mira	250		
CSEP025	Contig_c17429_mira	119		
CSEP026	Contig_c19570_mira	146		
CSEP027	Contig_c284_mira	202	70	bgh00979
CSEP028	Contig_c685_mira	418	65.13	bgh04235
CSEP029	Contig6408	88		
CSEP030	Contig_c15697_mira	179	45.37	bgh02588
CSEP031	Contig4366	517	67.64	bgh02390
CSEP032	Contig3171	547	72.86	bgh00747
CSEP033	Contig2741	347	57.85	bgh02693
CSEP034	Contig1984	304		
CSEP035	Contig3285	620	54.35	bgh01040
CSEP036	Contig7702	377		
CSEP037	Contig4347	818		
CSEP038	Contig_c2338_mira	385		
CSEP039	Contig1816	493		
CSEP040	5162_euler	353		
CSEP041	Contig770	208		
CSEP042	Contig7536	271		
CSEP043	Contig4219	237		
CSEP044	Contig_c23120_mira	68		
CSEP045	Contig3131	457		
CSEP046	Contig_c7631_mira	102		
CSEP047	Contig4562	763		
CSEP048	H36XR7X01A8UO1	129		
CSEP049	Contig_c14665_mira	245		
CSEP050	Contig_c11374_mira	184		
CSEP051	Contig4289	275		
CSEP052	Contig5572	162		
CSEP053	Contig5347	201		

An interesting group of annotated proteins is that composed of proteins related to host interaction (Table 2), formed by 17 predicted proteins. The amino acid sequence of these proteins is very similar to that of a protein of the barley powdery mildew *Blumeria graminis* f. sp. *hordei (Bgh)*, *Egh16H* [GenBank: Q9C1F7], which has been previously demonstrated to be upregulated in germinating conidia and during the formation of the primary infection structures on the host [30]. All of the proteins exhibit a DUF domain (Domain of Unknown Function); therefore, the biological function of these proteins remains to be determined.

In addition, six predicted proteins involved in pathogenesis (Table 2) were found in the secretome. One of them (contig2835) encodes a protein homologue of cerato-platanin (CP), a protein identified in *Ceratocystis fimbriata*, the causal agent of ‘‘canker stain disease” in plane trees, and involved in disease onset [31]. Three genes encoding putative secreted proteins containing CFEM domains (Common in Fungal Extracellular Membrane), which are fungi-specific, measure ~60 amino acids long, contain eight characteristically spaced cysteine residues and are believed to play roles in fungal pathogenesis [32]. One of the proteins (contig8430) is similar (48%) to the effector protein EC4 in *Bgh* [GenBank: AEQ16462). Another gene (contig3096) is similar (47%) to a *Fusarium verticillioides* 7600 protein [GenBank: EWG37445], which also carries a CFEM domain. The last protein (contig2707) is similar (37%) to an effector candidate previously identified in *Golovinomyes orontii*, EC2 [GenBank: G9BES2]. Another predicted secreted protein (contig7324) containing the CAP domain (Cysteine-rich secretory proteins, Antigen 5 and Pathogenesis-related protein) is similar (46%) to a *Colletotrichum graminicola* [GenBank: E3QP32] protein. The CAP superfamily proteins are found in all kingdoms of life and have been implicated in a variety of physiological contexts. In fungi, recent evidence indicates that the proteins act as sterol-binding and export proteins. The presence of these proteins in the secretome of pathogenic fungi suggests that they might inflict damage by sequestering sterols or related small hydrophobic compounds from the host tissue [33]. Finally, within the group of pathogenesis-related genes, a protein containing a glyoxal oxidase domain (contig1920) was found. This protein was similar (77%) to a protein identified in *Bgh* [GenBank: CCU82032], which is required for pathogenic development and cell morphology in *Ustilago maydis* [34].

Furthermore, one pathogenesis-related gene that does not have any predicted signal peptides was also identified. The gene is that of a putative RNA binding protein (contig310) that exhibits up to seven KH (K homology) domains and is related to RNA-binding proteins similar to the hypothetical effector protein Scp160, which has been identified in several pathogens, such as *Aspergillus fischerianus* and *Glomerella graminicola* [35].

### Identification of *P*. *xanthii* candidate effectors

**Fig 4:**
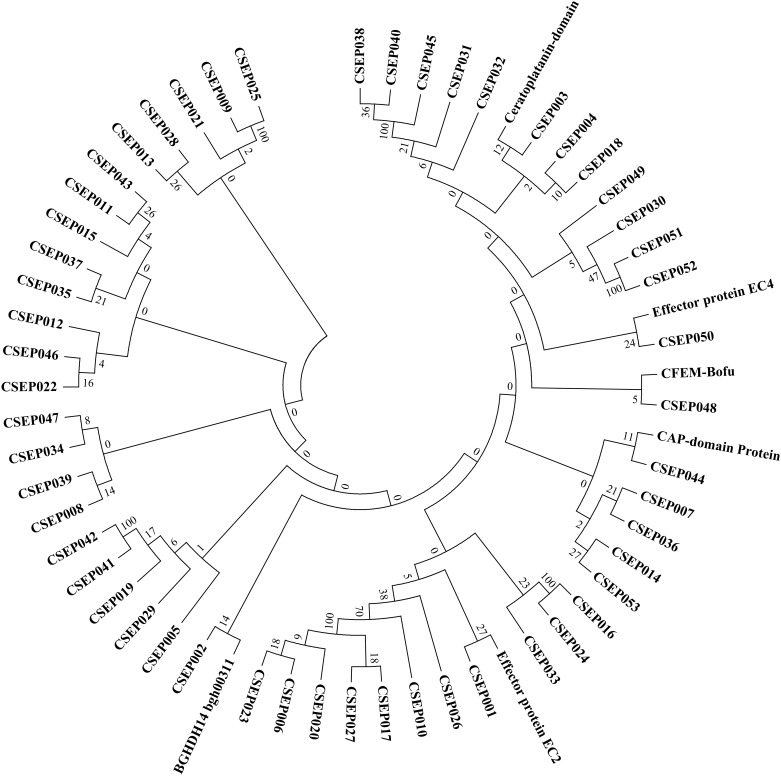
Phylogenetic analysis of *P*. *xanthii* CSEPs. The unrooted phylogenetic tree is based on NJ analysis. The confidence of groupings was estimated by using 1,000 bootstrap replicates, and the percentage of replicate trees supporting each branch is shown next to the branching point. The analysis showed the existence of potential families within the *P*. *xanthii* CSEPs.

In our analysis, we predicted 53 secreted proteins with no similarity with proteins in the public databases (Table 3); the proteins were referred to as candidate secreted effector proteins (CSEPs), which as suggested for other phytopathogenic fungi may be involved in pathogenesis. To test any existing relationship within the CSEPs group, among the CSEPs and with known effector proteins, an unrooted phylogenetic tree was constructed by the neighbour-joining method and bootstrap analysis among the 53 CSEPs specific to *P*. *xanthii* and 6 homologous effector proteins, mentioned above, identified in other filamentous fungi. Based on the phylogenetic analysis, all the putative secreted proteins were grouped into several families with two to six members (Fig 4), where the homologous proteins were spread all over the families and mixed with the CSEPs. Because of the high sequence diversity among the CSEPs, this approach did not accurately resolve their phylogenetic relationships but rather visualised clusters of similar sequences within the CSEP superfamily. Bootstrap analysis indicated largely reliable family classification, whereas the relatedness among families was less well determined.

**Fig 5:**
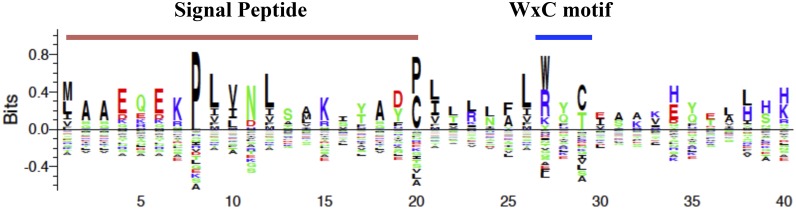
N-terminal amino acid Y/F/WxC motif and natural selection in *P*. *xanthii* CSEPs. The N-terminal amino acid motif Y/F/WxC was screened manually within the pool of CSEPs, and only the motif WxC was observed following the secretion signal. Alignments were performed with ClustalW2, and the sequence logo was represented using the Seq2Logo online server (www.cbs.dtu.dk/biotools/Seq2Logo/).

Furthermore, manual inspection of the alignment revealed that *P*. *xanthii* CSEPs contained the N-terminal conserved motif Y/F/WxC, previously found in many candidate effector proteins in powdery mildew fungi [10]. Notably, the WxC motif was particularly abundant (Fig 5). In addition, the putative transcriptome of *P*. *xanthii* was compared with the EST collection of *Bgh* (www.blugen.org) by BlastP analysis. Only 44% of genes (3,908 genes) were common between *P*. *xanthii* and *Bgh*. The group of common genes was used as database to map the CSEPs identified in *P*. *xanthii*; 17 of 53 CSEPs were shared with *Bgh*, whereas the rest were specific to *P*. *xanthii*. The *P*. *xanthii* CSEPs that are also present in *Bgh* are indicated in Table 3.

### Expression analysis of *P*. *xanthii* CSEPs

**Fig 6:**
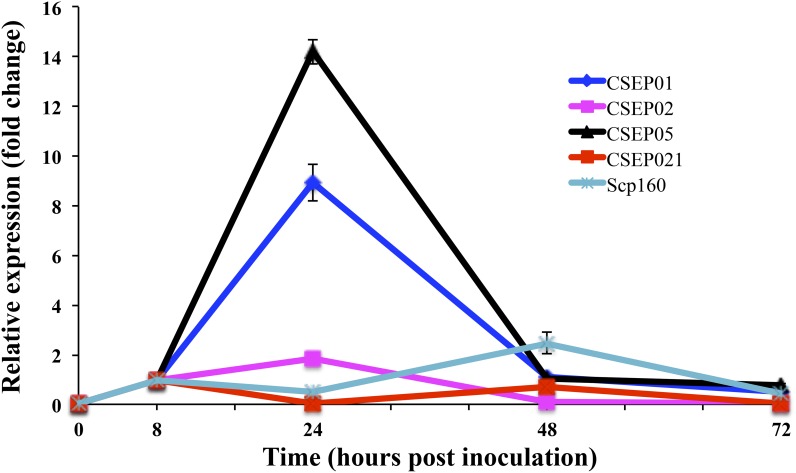
Expression of selected *P*. *xanthii* CSEP genes in a time-course experiment. Total RNA was isolated from zucchini cotyledons infected with *P*. *xanthii* at different time points, and the relative expression of five putative effector-genes (*CSEP01*, *CSEP02*, *CSEP05*, *CSEP021* and a homologue to *Scp160*) was analysed by quantitative RT-PCR. Transcript abundance was normalised to the endogenous control β-tubulin gene *PxTUB*2 (KC333362). Relative expression of each gene was calibrated to the sample 8 h post inoculation. Points represent the mean ± standard deviation of three technical replicates from two cDNA samples obtained from two different zucchini cotyledons.

To test whether the CESPs identified in this work could play a role during interaction with host plants, time-course transcriptional profiling of a small group of selected *P*. *xanthii* CSEPs was performed. These selected genes include three *P*. *xanthii*-specific CSEPs (CSEP01, CSEP02 and CSEP05), and two additional CESPs with putative orthologues in other phytopathogenic fungi, CSEP021 (contig8160) a homologue of a hypothetical protein of *B*. *graminis* which encodes a putative peptidase, and the putative RNA binding protein (contig310) homologue to the hypothetical effector protein Scp160 identified in *A*. *fischerianus* and *G*. *graminicola*. RT-qPCR was carried out to analyse the expression pattern of these genes (Fig 6). Three of five CSEPs screened (CSEP01, CSEP02 and CSEP05) showed maxima of expression at the beginning of the infection process (24 hpi), when the primary appressoria are mostly formed. CSEP01 and CSEP05 showed 9-fold and 14-fold increases, respectively, whereas a slight twofold increase was observed for CSEP02. The homologue to the effector Scp160 (contig 310) displayed a delay in the expression pattern with a maximum of twofold increase at 48 hpi, when primary hyphae are abundant. By contrast, expression of CSEP021 hardly changed over time. After 48 hpi, the analysed candidate effectors showed expression levels very reduced (one-fold change or less).

## Discussion

The cucurbit family includes many economically important species, particularly those with edible fruits. There are over 200 known cucurbit diseases of diverse aetiologies; however, powdery mildew is likely the most common, conspicuous, widespread and easily recognisable disease in these crops [14]. Despite the agronomic and economic relevance of the fungus, no genomic resources and few molecular tools are available for studying this fungus. The genomes of powdery mildew fungi appear to be very complex, not only because of their size (approximately 120 Mbp, nearly 4 times the size of most other ascomycetes) but also because of the high abundance of repetitive DNA, particularly retroelements (approximately 70% of the genome) [3–5]. To obtain large-scale genome sequence information from *P*. *xanthii*, we needed a strategy to reduce genome complexity. For this purpose, we chose cDNA sequencing for gene discovery. Indeed, these results could be used for genome annotation in future genome sequencing projects.

Here, we used 454 GS FLX (Roche) to sequence a cDNA library from epiphytic mycelium and conidia of *P*. *xanthii* and thereby characterise the transcripts. We identified 6,645 genes in *P*. *xanthii*. This number of genes identified in *P*. *xanthii* is very similar to the gene number described previously for *B*. *graminis*, with 6,470 annotated genes [3]. This similarity means that our assembly is likely to encompass most of the genes and represent an unbiased fraction of the total transcriptome of *P*. *xanthii*. In contrast to previous studies in which Blast2GO [36] was the most common bioinformatics tool employed to annotate the transcripts [37,38], in this work we used three different annotation programs FulLengtherNext [39], AutoFACT [23] and Sma3s [22], thus maximising the yield in gene identification.

In previous studies, transcriptomic analyses of powdery mildew fungi have shown that *B*. *graminis* and *G*. *orontii* contain many transcripts involved in primary metabolic process and protein synthesis as well as pathogenesis [38,40–42]. Similarly, among the genes associated with protein metabolism, most of the genes present in *P*. *xanthii* transcriptome are related to protein synthesis and protein modification processes. In addition, vital cellular processes such as the regulation of biological processes and organelle organisation are also highly represented in the *P*. *xanthii* transcriptome, as recently shown for *Podosphaera plantaginis* [43]. Moreover, components of protein biosynthesis are highly overrepresented in transcripts of ascomycete phytopathogens compared with those of non-pathogenic ascomycete fungi [44], indicating a general role of high translational activity during adaption to the host environment.

By comparison with EST collections, proteomes and genomes from other ascomycetes, we identified metabolic pathways missing in *P*. *xanthii* as well as in other powdery mildews [3]. The missing genes are involved in pathways associated with a saprophytic life-style. One of these pathways is thiamine biosynthesis; thiamine diphosphate (vitamin B1) is a cofactor required for the activity of several enzymes of central carbon metabolism, such as pyruvate dehydrogenase, pyruvate decarboxylase, α-ketoglutarate decarboxylase, and transketolase [45]. Another absent group of genes is related to allantoin metabolism; allantoin is an intermediate in the degradation of adenine and guanine and is a major nitrogen source for *S*. *cerevisiae* growing in its natural habitat [46]. Thus, powdery mildew fungi are not capable of using allantoin as a nitrogen source. Moreover, powdery mildews lack genes involved in inorganic nitrogen assimilation because there is no trace of genes related to methionine metabolism and nitrate assimilation, both of which are pathways responsible for nitrogen assimilation for protein synthesis [47,48].

Furthermore, *P*. *xanthii* also lacks the repeat-induced point mutation (RIP) systems as in *B*. *graminis* and *G*. *orontii* [3]. The RIP mechanism was described for the first time for the ascomycete fungus *Neurospora crassa* and is responsible for duplicated gene removal during meiosis, therefore, limiting the ability to undergo paralogous gene duplication and consequent gene family expansion [49]. Moreover, the RIP system is widely believed to protect fungal genomes against transposon replication [50,51] and therefore appears to contribute to the evolution of fungal genes. This system has the potential to either enhance or impede the generation of genetic diversity. In a previous study, authors described that high levels of RIP lead to a deficiency in gene family diversity, whereas low levels of RIP lead to increased diversity of gene families. Furthermore, RIP has been reported to affect non-duplicated genes adjacent to RIP-affected repetitive DNA sequences, which may drive the evolution of these genes and can promote rapid adaptation to selection pressures in some species [52].

The secretion of proteins and metabolites that mediate the communication between host and pathogen are termed effectors. Effector proteins play a pivotal role in determining the outcome of the interactions [24]. Although the specific processes for host colonisation are mediated by the haustorium [38], a number of different effector candidates were detected in the epiphytic transcriptome. We found a number of transcripts encoding secreted cell wall-modifying enzymes, such as putative chitinases, carboxylesterases, carboxypeptidases and two glucanosyltransferases. The latter two transcripts are homologues of *GAS1*, that encodes for a protein involved in fungal cell wall biogenesis and described as a virulence factor of *Fusarium oxysporum* [53]. This gene has also been identified in *B*. *graminis* and implicated in virulence by transient silencing assays [54]. We also identified several homologues of other pathogenicity genes. For example, a homologue of a cerato-platanin (CP) protein of *Ceratocystis fimbriata* f. sp. *platani*, the causal agent of canker stain disease [31], was identified. CP shares certain structural and functional characteristics with other fungal proteins called hydrophobins that are secreted fungal proteins and have a wide range of functions in fungal growth and development. These proteins are able to self-assemble, resulting in large macrofibrillar assemblies, and bind to hydrophobic/hydrophilic interfaces acting as biosurfactants [55]. Interestingly, there are no hydrophobin genes in the powdery mildew fungi whose genomes have been analysed to date. One of these proteins, the product of the *snodprot1* gene from *Phaeosphaeria nodorum*, the causal agent of glume blotch of wheat, is expressed during infection of wheat leaves and appears to be involved in various stages of the host-fungus interaction [31].

We found three transcripts that contain the cysteine-rich domain (CFEM), also detected in many fungal effector proteins described previously in the secretome of *Sclerotinia sclerotiorum* [56]. One of these proteins is a possible homologue of the *G*. *orontii EC2* gene, which encodes another putative effector protein. Indeed, two proteins containing this motif, Pth11 and ACI1, appear to play an important role in appressorium development and cAMP production, respectively, in the rice blast fungus *Magnaporthe grisea* [57,58]. It has been suggested that CFEM-domain proteins could function as cell-surface receptors or signal transducers or as adhesion molecules in host-pathogen interactions [32]. We also found one protein belonging to the CAP protein family (cysteine-rich secretory proteins, antigen 5 and pathogenesis-related 1 proteins), which are involved in many processes including the regulation of extracellular matrix and branching morphogenesis and potentially as either proteases or protease inhibitors, among others [59].

In addition, a putative gene encoding a protein similar to the Scp160 effector protein of *A*. *fischerianus* was found [35]; this protein contains a KH-1 domain that is present in a wide variety of nucleic acid-binding proteins. The KH domains are implicated in mRNA binding and have been described as responsible for mRNA degradation [60]. Furthermore, a transcript that encodes for a putative glyoxal oxidase was also found in the *P*. *xanthii* transcriptome. This enzyme catalyses the enzymatic oxidation of a variety of simple dicarbonyl and β-hydroxycarbonyls, particularly glyoxal and methylglyoxal, to carboxylic acids, coupled with hydrogen peroxide production. In *U*. *maydis*, the authors demonstrated that the membrane-bound glyoxal oxidase Glo1 is required for pathogenic development and for the maintenance of cell morphology and filamentous growth [34].

As observed in the barley powdery mildew *B*. *graminis* f. sp. *hordei* [6], the CSEPs identified in *P*. *xanthii* showed a certain type of relationship among them and with known effector proteins. The clustering of CSEPs into families of paralogues suggests that CSEPs have gone through iterated rounds of gene duplication during evolution [10], and those local duplications might be involved in the expansion of effectors in *P*. *xanthii*, as described previously for *Ustilaginoidea virens* [61]. Despite no functional explanation for such clustering, the relationship shared with known effector proteins could be used to assign a possible functional categorisation to those CSEPs that cluster.

The number of total CSEPs obtained in the present study was lower than that observed in other powdery mildew transcriptomic studies [6,10,38]. However, those studies focused on effector candidates obtained from the specialised feeding structure, the haustorium [51], and not on surface structures such as mycelia and conidia. Therefore, because the haustorium is the structure that is intimately related to the host plant, it is reasonable that these specialised cells express a higher number of effector proteins than the epiphytic mycelium does. In any case, the expression profile of some of these CSEPs resembles those previously observed in other powdery mildew species for candidate effectors [38], suggesting a role of these CSEPs during the pathogenic process. Finally, *P*. *xanthii* CSEPs were screened for the typical N-terminal conserved motif Y/F/WxC-motif [10]; interestingly, the WxC motif was predominant in epiphytic effector candidates. The preference for this motif as well as its biological significance in *P*. *xanthii* mycelium-associated effectors remains unknown.

*Podosphaera xanthii*, like the rest of powdery mildew fungi, is an obligate biotroph. This means, it is able to grow on plants by obtaining nutrients from host cells that are still alive. To accomplish this goal, the pathogen has to cope with PTI (PAMP-triggered immunity), a defense response which is induced by fungal contact with the plant surface. The effector armory of biotrophs includes effectors needed during penetration to downregulate PTI [8]. Some of these effectors have been described in model biotrophs such as *U*. *maydis*, *Cladosporium fulvum* or *B*. *graminis* f. sp. *hordei*. To identify similar effectors in *P*. *xanthii*, specific tools for functional validation of candidate effector genes should be previously developed. With this regard, the host-induced gene silencing (HIGS) technique [54] is currently under development in our laboratory.

## Conclusions

The present work provides transcriptome information on the cucurbit powdery mildew pathogen *P*. *xanthii*. Insight into the molecular bases of obligate biotrophy and pathogenesis, completely unknown to date, have been provided. Our results represent a source of molecular information that can be used for a number of downstream applications in different fields such as epidemiology and population genetics, systematics, fungicide resistance or functional genomics. With respect to functional genomics, however, specific tools that allow for systematic functional validation of genes involved in pathogenesis and other biological processes in this important pathogen of cucurbits should be developed.

## Supporting Information

S1 Fig Schematic workflow used for assembly of the epiphytic transcriptome of *P*. *xanthii*. See Materials and Methods for details.
(TIF)

S2 Fig Schematic workflow used to identify the epiphytic secretome of *P*. *xanthii* including candidate secreted effector proteins (CSEPs). See Materials and Methods for details.
(TIF)

S1 Table Primers used for time-course gene expression profiling experiments. (DOCX)

S2 Table Top50 most expressed transcripts in the *P*. *xanthii* epiphytic transcriptome. (DOCX)

S3 Table Metabolic pathways missing in *P*. *xanthii* as in other powdery mildews. (DOCX)

S4 Table Annotation of the *P*. *xanthii* epiphytic secretome (rest of categories). Categories “Pathogenesis” and “Host interaction”, and the list of CSEPs are shown in Tables 2 and 3, respectively.
(DOCX)
