# Supplementary figures and images for: De novo Analysis of the Epiphytic Transcriptome of the Cucurbit Powdery Mildew Fungus Podosphaera xanthii and Identification of Candidate Secreted Effector Proteins
Source: PLoS One. 2016 Oct 6;11(10):e0163379. doi: 10.1371/journal.pone.0163379 (PMC5053433; doi:10.1371/journal.pone.0163379)

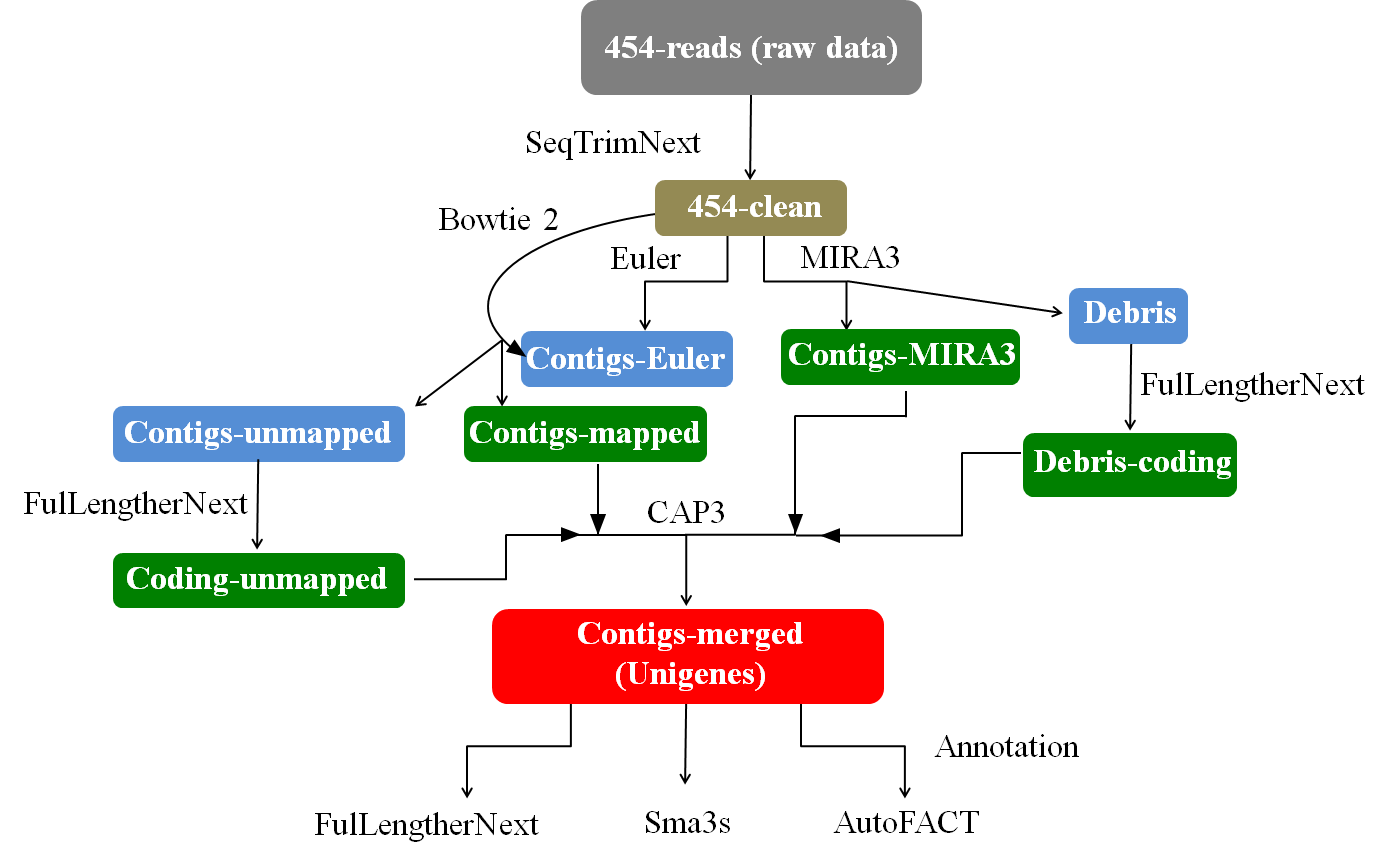

Supplement: S1 Fig — See Materials and Methods for details. (TIF) [file pone.0163379.s001.tif]

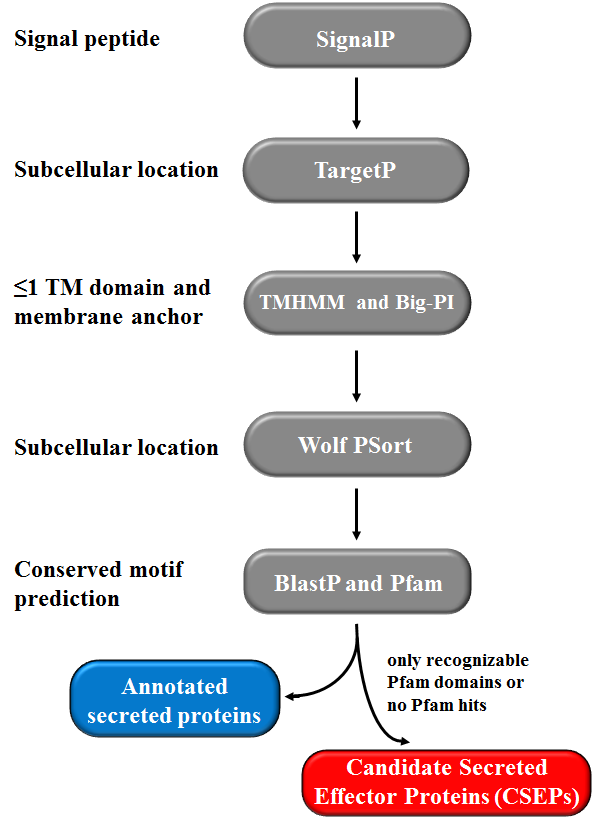

Supplement: S2 Fig — See Materials and Methods for details. (TIF) [file pone.0163379.s002.tif]
