# Supplementary material for: De novo Analysis of the Epiphytic Transcriptome of the Cucurbit Powdery Mildew Fungus Podosphaera xanthii and Identification of Candidate Secreted Effector Proteins
Source: PLoS One. 2016 Oct 6;11(10):e0163379. doi: 10.1371/journal.pone.0163379 (PMC5053433; doi:10.1371/journal.pone.0163379)
Supplement: S1 Table — (DOCX) [file pone.0163379.s003.docx]

| **S1 Table. Primers used for time-course gene expression profiling experiments.** | | |
| --- | --- | --- |
| **Target gene** | **Primer name** | **Sequence (5’-3’)** |
| *TUB2* | tub2-F | CAAGTCGTGGTGCCCATTCT |
|  | tub2-R | GGTCGAACATCTGTTGGGTTAATT |
| *Scp160* | Scp160-F | TGTCCGTGTCTTCGTGTCTGG |
|  | Scp160-R | CGTCTTCGAATGCCTGCTCCA |
| *CSEP01* | CSEP01-F | GAGCATCCTCGATGCGGCTGA |
|  | CSEP01-R | GCTCACCGGCTGCCAATTCCA |
| *CSEP02* | CSEP02-F | TGTTCGGCGGCGACATGACC |
|  | CSEP02-R | GAGGCGAGAGCCTGCCGAGA |
| *CSEP05* | CSEP05-F | CAGGTGCCCGCCGATCTTCC |
|  | CSEP05-R | GACTCGGTGGCGCGATCAGG |
| *CSEP021* | CSEP021-F | TGTTTGCCGGGAAGCCAGACG |
|  | CSEP021-R | AGGGGTCGCCCGCAAAGCAG |
