# Supplementary material for: De novo Analysis of the Epiphytic Transcriptome of the Cucurbit Powdery Mildew Fungus Podosphaera xanthii and Identification of Candidate Secreted Effector Proteins
Source: PLoS One. 2016 Oct 6;11(10):e0163379. doi: 10.1371/journal.pone.0163379 (PMC5053433; doi:10.1371/journal.pone.0163379)
Supplement: S2 Table — (DOCX) [file pone.0163379.s004.docx]

| TABLE S2 Top50 most expressed transcripts in the epiphytic transcriptome of *P. xanthii*. | | | | | | | |
| --- | --- | --- | --- | --- | --- | --- | --- |
| **Sequence ID** | **Reads number** | | **Description** | **E-value^a^** | **Sequence identity (%)** | **Subject ID^b^** | **Protein length (aa)** |
| **Translation** |  | |  |  |  |  |  |
| Contig3336 | 2591 | | Elongation factor 1-alpha | 0.0 | 92% (407/438) | Q01520 | 460 |
| Contig2743 | 1518 | | 40S ribosomal protein S3aE | 1.00E-146 | 62% (257/414) | P36008 | 415 |
| Contig3198 | 2161 | | Woronin body major protein | 6.00E-78 | 41% (178/432) | P87252 | 171 |
| Contig8845 | 605 | | Ribosomal protein | 1.00E-117 | 87% (213/244) | P29453 | 265 |
| Contig4895 | 1677 | | 60S ribosomal protein L7 | 2.00E-97 | 75% (173/230) | A6SRU1 |  |
| Contig2711 | 1510 | | Eukaryotic translation initiation factor subunit eIF2A | 0.0 | 69% (355/509) | O74965 | 530 |
| Contig8729 | 870 | | Ribosomal protein | 1.00E-101 | 87% (190/217) | Q7RZS0 | 218 |
| Contig6894 | 627 | | 40S ribosomal protein S2 | 1.00E-109 | 89% (202/226) | P25443 | 261 |
| Contig2723 | 483 | | 60S ribosomal protein L15 | 8.00E-99 | 87% (178/203) | Q8X034 | 204 |
| **Protein modification process** | | | | |  |  |  |
| Contig5914 | 1509 | | Serine protease | 1.00E-173 | 65% (302/460) | C5P4Z8 | 464 |
| Contig2714 | 1089 | | Aspartyl protease | 1.00E-131 | 58% (269/463) | Q4WZS3 | 483 |
| **Energy metabolism** | | | |  |  |  |  |
| Contig2753 | 900 | | Glyceraldehyde-3-phosphate dehydrogenase | 1.00E-156 | 81% (272/335) | Q96US8 | 339 |
| Contig6433 | 949 | | Pyruvate carboxylase | 0.0 | 81% (977/1206) | Q9HES8 | 1193 |
| Contig3899 | 528 | | ATP synthase subunit beta | 0.0 | 91% (462/507) | P23704 | 511 |
| Contig1987 | 505 | | Pyruvate kinase | 0.0 | 76% (224/294) | P46614 | 528 |
| Contig2709 | 1758 | | Dihydrofolate reductase | 6.00E-44 | 38% (116/300) | A1CJD1 |  |
| **Pathogenesis** |  | |  |  |  |  |  |
| Contig8658 | 617 | | CAP20-like protein | 5.00E-98 | 74% (141/190) | Q96WJ9 | 237 |
| Contig2707 | 1090 | | Effector protein EC2 | 1.00E-11 | 37% (26/70) | G9BES2 | 181 |
| 11744_euler | 852 | | CAP20-like protein | 2.00E-89 | 69% (134/194) | Q96WJ9 | 229 |
| **Catabolic process** | | | |  |  |  |  |
| Contig8559 | 841 | | Peptidase C1-like family protein | 1.00E-176 | 60% (313/519) | E3Q7I1 | 502 |
| Contig2338 | 460 | | Superoxide dismutase | 9.00E-79 | 65% (142/216) | Q9Y783 | 217 |
| Contig1986 | 461 | | 1,3-beta-glucanosyltransferase | 1.00E-178 | 73%  (311/424) | P0C7S9 | 448 |
| Contig_c5406_mira | 445 | | Dihydrofolate reductase | 3.00E-44 | 38% (116/300) | G2Y8R7 | 369 |
| **Reproduction** |  | |  |  |  |  |  |
| Contig2960 | 770 | | Mating_C protein | 1.00E-04 | 23%  (32/135) | E5A5H0 | 172 |
| **Transport** |  | |  |  |  |  |  |
| Contig2722 | 826 | | MFS drug efflux pump | 6.00E-86 | 32% (185/566) | EAW15529 | 689 |
| Contig2777 | 614 | | Cobalamin-independent methionine synthase | 0.0 | 79% (616/774) | CBF77508 | 768 |
| Contig5295 | 803 | | Similar to vacuolar calcium ion transporter /H(+) exchanger | 1.00E-123 | 59% (238/400) | E4ZJ94 | 445 |
| Contig2773 | 449 | | MFS transporter | 2.00E-91 | 36% (193/536) | Q10084 | 530 |
| **No functional annotation** | | | |  |  |  |  |
| Contig2719 | 491 | | Putative uncharacterized protein | 5.00E-11 | 20% (26/125) | G2YJF9 | 250 |
| Contig8286 | 1240 | | Unassigned protein |  |  |  |  |
| Contig2762 | 521 | | Unassigned protein |  |  |  |  |
| Contig4616 | 716 | | uncharacterized protein |  |  |  |  |
| Contig_c33_mira | 720 | | Unassigned protein |  |  |  |  |
| Contig2729 | 567 | | uncharacterized protein |  |  |  |  |
| Contig_c2039_mira | 854 | | Unassigned protein |  |  |  |  |
| Contig2724 | 663 | | Unassigned protein |  |  |  |  |
| Contig9103 | 870 | | Putative Egh16H1 isoform | 1.00E-137 | 76% (243/319) | Q9C1F6 | 316 |
| Contig_c21_mira | 633 | | Unassigned protein |  |  |  |  |
| Contig4709 | 469 | | Unassigned protein |  |  |  |  |
| Contig2886 | 468 | | Putative uncharacterized protein | 3.00E-44 | 43%  (87/201) | A7EU44 | 203 |
| Contig2757 | 456 | | Putative BIG4 | 1E-109 | 53% (202/375) | Q6QT09 | 377 |
| Contig1984 | 441 | | Putative uncharacterized protein | 4E-05 | 22%  (54/241) | G2YKE9 | 304 |
| **Cell cycle** |  | |  |  |  |  |  |
| Contig3439 | 596 | | Carnitine acetyl transferase | 0.0 | 76% (481/636) | EMR81316 | 636 |
| **Nitrogen compound metabolic process** | | | | |  |  |  |
| Contig2715 | 594 | | ATP synthase protein 9 | 7.00E-32 | 55% (87/157) | P16000 | 158 |
| **Secondary metabolism** | | | |  |  |  |  |
| Contig3302 | 544 | | Carboxypeptidase Y homolog A | 0.0 | 69% (372/537) | A7F4H5 | 530 |
| Contig_c41_mira | 448 | | Peroxisomal hydratase-dehydrogenase-epimeras | 0.0 | 74% (443/596) | Q01373 | 605 |
| **Cell wall organization** | | | |  |  |  |  |
| Contig2735 | 502 | | Glucan 1,3-beta-glucosidase | 1.00E-138 | 58% (235/405) | G2XSV1 | 419 |
| **RNA metabolism** | | | |  |  |  |  |
| Contig2733 | 493 | | Tyrosinase | 1.00E-83 | 33% (201/595) | B8MQC5 | 584 |
| **Protein catabolism** | | | |  |  |  |  |
| Contig7749 | | 473 | Succinate dehydrogenase subunit A | 0.0 | 86% (560/645) | B8XCQ0 | 649 |
| **Signal transduction** | |  |  |  |  |  |  |
| Contig7458 | | 463 | Putative Phosducin-like protein | 7E-60 | 45% (133/293) | H0ECS3 | 282 |
| ^a^ E-values were obtained after Blast analysis | | | | | | | |
| ^b^GenBank accession number | | | | | | | |
