# Supplementary material for: De novo Analysis of the Epiphytic Transcriptome of the Cucurbit Powdery Mildew Fungus Podosphaera xanthii and Identification of Candidate Secreted Effector Proteins
Source: PLoS One. 2016 Oct 6;11(10):e0163379. doi: 10.1371/journal.pone.0163379 (PMC5053433; doi:10.1371/journal.pone.0163379)
Supplement: S3 Table — (DOCX) [file pone.0163379.s005.docx]

| **S3 Table. Metabolic pathways missing in *P. xanthii* as in other powdery mildews.** | | | | | |
| --- | --- | --- | --- | --- | --- |
| **Systematic name** | **Gene name** | **Description** | ***S. cerevisiae* (proteome)** | ***B. graminis* (genome)** | ***P. xanthii* (EST)** |
| **Thiamine metabolism/transport** | | | | | |
| YGR144W | THI4 | Thiazole synthase, catalyzes formation of a thiazole intermediate during thiamine biosynthesis; required for mitochondrial genome stability in response to DNA damaging agents. | + | - | 7.00E-86 |
| YPL214C | THI6 | Bifunctional enzyme with thiamine-phosphate pyrophosphorylase and 4-methyl-5-beta-hydroxyethylthiazole kinase activities, required for thiamine biosynthesis; GFP-fusion protein localizes to the cytoplasm in a punctate pattern. | + | - | - |
| YLR237W | THI7 | Plasma membrane transporter responsible for the uptake of thiamine, member of the major facilitator superfamily of transporters; mutation of human ortholog causes thiamine-responsive megaloblastic anemia. | + | - | - |
| YOL055C | THI20 | Multifunctional protein with hydroxymethylpyrimidine phosphate (HMP-P) kinase and thiaminase activities; involved in thiamine biosynthesis and degradation; in a gene family with THI21 and THI22; HMP-P kinase activity redundant with Thi21p. | + | - | - |
| YPL258C | THI21 | Hydroxymethylpyrimidine phosphate kinase, involved in the last steps in thiamine biosynthesis; member of a gene family with THI20 and THI22; Thi20p also has this activity. | + | - | - |
| YPR121W | THI22 | Protein with similarity to hydroxymethylpyrimidine phosphate kinases; member of a gene family with THI20 and THI21; not required for thiamine biosynthesis. | + | - | - |
| YOR192C | THI72 | Transporter of thiamine or related compound; shares sequence similarity with Thi7p. | + | - | - |
| YOR071C | NRT1 | High-affinity nicotinamide riboside transporter; also transports thiamine with low affinity; shares sequence similarity with Thi7p and Thi72p; proposed to be involved in 5-fluorocytosine sensitivity. | + | - | - |
| **Allantoine metabolism/transport** | | | | | |
| YIR027C | DAL1 | Allantoinase, converts allantoin to allantoate in the first step of allantoin degradation; expression sensitive to nitrogen catabolite repression. | + | - | - |
| YIR029W | DAL2 | Allantoicase, converts allantoate to urea and ureidoglycolate in the second step of allantoin degradation; expression sensitive to nitrogen catabolite repression and induced by allophanate, an intermediate in allantoin degradation. | + | - | - |
| YIR028W | DAL4 | Allantoin permease; expression sensitive to nitrogen catabolite repression and induced by allophanate, an intermediate in allantoin degradation. | + | - | - |
| YIR023W | DAL81 | Positive regulator of genes in multiple nitrogen degradation pathways; contains DNA binding domain but does not appear to bind the dodecanucleotide sequence present in the promoter region of many genes involved in allantoin catabolism. | + | - | - |
| YHL016C | DUR3 | Plasma membrane transporter for both urea and polyamines, expression is highly sensitive to nitrogen catabolite repression and induced by allophanate, the last intermediate of the allantoin degradative pathway. | + | - | - |
| **Methionine metabolism and (siro-)heme biosynthesis** | | | | | |
| YKR069W | MET1 | S-adenosyl-L-methionine uroporphyrinogen III transmethylase, involved in the biosynthesis of siroheme, a prosthetic group used by sulfite reductase; required for sulfate assimilation and methionine biosynthesis. | + | - | - |
| YJR010W | MET3 | ATP sulfurylase, catalyzes the primary step of intracellular sulfate activation, essential for assimilatory reduction of sulfate to sulfide, involved in methionine metabolism. | + | - | - |
| YBR213W | MET8 | Bifunctional dehydrogenase and ferrochelatase, involved in the biosynthesis of siroheme, a prosthetic group used by sulfite reductase; required for sulfate assimilation and methionine biosynthesis. | + | - | - |
| YKL001C | MET14 | Adenylylsulfate kinase, required for sulfate assimilation and involved in methionine metabolism. | + | - | - |
| YPR167C | MET16 | 3'-phosphoadenylsulfate reductase, reduces 3'-phosphoadenylyl sulfate to adenosine-3',5'-bisphosphate and free sulfite using reduced thioredoxin as cosubstrate, involved in sulfate assimilation and methionine metabolism. | + | - | - |
| YOR278W | HEM4 | V from 842816-843643, Verified ORF, "Uroporphyrinogen III synthase, catalyzes the conversion of hydroxymethylbilane to uroporphyrinogen III, the fourth step in heme biosynthesis; deficiency in the human homolog can result in the disease congenital erythropoietic porphyria". | + | - | - |
| **Alcohol metabolism/fermentation** | | | | | |
| YGL256W | ADH4 | Alcohol dehydrogenase isoenzyme type IV, dimeric enzyme demonstrated to be zinc-dependent despite sequence similarity to iron-activated alcohol dehydrogenases; transcription is induced in response to zinc deficiency. | + | - | - |
| YCR107W | AAD3 | Putative aryl-alcohol dehydrogenase with similarity to P. chrysosporium aryl-alcohol dehydrogenase; mutational analysis has not yet revealed a physiological role. | + | - | - |
| YDL243C | AAD4 | Putative aryl-alcohol dehydrogenase with similarity to P. chrysosporium aryl-alcohol dehydrogenase, involved in the oxidative stress response; expression induced in cells treated with the mycotoxin patulin. | + | - | - |
| YFL056C | AAD6 | Putative aryl-alcohol dehydrogenase with similarity to P. chrysosporium aryl-alcohol dehydrogenase, involved in the oxidative stress response; expression induced in cells treated with the mycotoxin patulin. | + | - | - |
| YJR155W | AAD10 | Putative aryl-alcohol dehydrogenase with similarity to P. chrysosporium aryl-alcohol dehydrogenase; mutational analysis has not yet revealed a physiological role. | + | - | - |
| YNL331C | AAD14 | Putative aryl-alcohol dehydrogenase with similarity to P. chrysosporium aryl-alcohol dehydrogenase; mutational analysis has not yet revealed a physiological role. | + | - | - |
| YOL165C | AAD15 | Putative aryl-alcohol dehydrogenase with similarity to P. chrysosporium aryl-alcohol dehydrogenase; mutational analysis has not yet revealed a physiological role. | + | - | - |
| YFL057C | AAD16 | Putative aryl-alcohol dehydrogenase with similarity to P. chrysosporium aryl-alcohol dehydrogenase; mutational analysis has not yet revealed a physiological role. | + | - | - |
| YPL088W | YPL088W | Putative aryl alcohol dehydrogenase; transcription is activated by paralogous transcription factors Yrm1p and Yrr1p along with genes involved in multidrug resistance | + | - | - |
| YLR044C | PDC1 | Major of three pyruvate decarboxylase isozymes, key enzyme in alcoholic fermentation, decarboxylates pyruvate to acetaldehyde; subject to glucose-, ethanol-, and autoregulation; involved in amino acid catabolism. | + | 1.00E-07 | - |
| YOL086C | ADH1 | Alcohol dehydrogenase, fermentative isozyme active as homo- or heterotetramers; required for the reduction of acetaldehyde to ethanol, the last step in the glycolytic pathway. | + | - | - |
| **Glutamate metabolism** | | | | | |
| YOR375C | GDH1 | NADP(+)-dependent glutamate dehydrogenase, synthesizes glutamate from ammonia and alpha-ketoglutarate; rate of alpha-ketoglutarate utilization differs from Gdh3p; expression regulated by nitrogen and carbon sources. | + | - | - |
| YAL062W | GDH3 | NADP(+)-dependent glutamate dehydrogenase, synthesizes glutamate from ammonia and alpha-ketoglutarate; rate of alpha-ketoglutarate utilization differs from Gdh1p; expression regulated by nitrogen and carbon sources. | + | - | - |
| **Uracil metabolism/transport** | | | | | |
| YBL042C | FUI1 | High affinity uridine permease, localizes to the plasma membrane; also mediates low but significant transport of the cytotoxic nucleoside analog 5-fluorouridine; not involved in uracil transport. | + | - | - |
| YBR021W | FUR4 | Uracil permease, localized to the plasma membrane; expression is tightly regulated by uracil levels and environmental cues. | + | - | - |
| YKL216W | URA1 | Dihydroorotate dehydrogenase, catalyzes the fourth enzymatic step in the de novo biosynthesis of pyrimidines, converting dihydroorotic acid into orotic acid. | + | - | - |
| **Glutathione metabolism** | | | | | |
| YGR154C | GTO1 | Omega-class glutathione transferase; induced under oxidative stress; putative peroxisomal localization. | + | - | - |
| YMR251W | GTO3 | Omega class glutathione transferase; putative cytosolic localization. | + | - | - |
| YKR076W | ECM4 | Omega class glutathione transferase; not essential; similar to Ygr154cp; green fluorescent protein (GFP)-fusion protein localizes to the cytoplasm. | + | - | - |
| YLR299W | ECM38 | Gamma-glutamyltranspeptidase, major glutathione-degrading enzyme; involved in detoxification of electrophilic xenobiotics; expression induced mainly by nitrogen starvation. | + | - | - |
| **Detoxification/stress response** | | | | | |
| YER185W | PUG1 | Plasma membrane protein with roles in the uptake of protoprophyrin IX and the efflux of heme; expression is induced under both low-heme and low-oxygen conditions; member of the fungal lipid-translocating exporter (LTE) family of proteins. | + | - | - |
| YGR213C | RTA1 | Protein involved in 7-aminocholesterol resistance; has seven potential membrane-spanning regions; expression is induced under both low-heme and low-oxygen conditions; member of the fungal lipid-translocating exporter (LTE) family of protein. | + | - | - |
| YLR046C | YLR046C | Putative membrane protein; member of the fungal lipid-translocating exporter (LTE) family of proteins; transcription is activated by paralogous transcription factors Yrm1p and Yrr1p along with genes involved in multidrug resistance. | + | - | - |
| YJR104C | SOD1 | Cytosolic copper-zinc superoxide dismutase; some mutations are analogous to those that cause ALS (amyotrophic lateral sclerosis) in humans. | + | - | - |
| YGR234W | YHB1 | Nitric oxide oxidoreductase, flavohemoglobin involved in nitric oxide detoxification; plays a role in the oxidative and nitrosative stress responses. | + | - | - |
| YIL053W | RHR2 | Constitutively expressed isoform of DL-glycerol-3-phosphatase; involved in glycerol biosynthesis, induced in response to both anaerobic and, along with the Hor2p/Gpp2p isoform, osmotic stress. | + | - | - |
| YPR201W | ARR3 | Arsenite transporter of the plasma membrane, required for resistance to arsenic compounds; transcription is activated by Arr1p in the presence of arsenite. | + | - | - |
| YGL196W | DSD1 | D-serine dehydratase (aka D-serine ammonia-lyase); converts D-serine to pyruvate and ammonia by a reaction dependent on pyridoxal 5'-phosphate and zinc; may play a role in D-serine detoxification; L-serine is not a substrate. | + | - | - |
| YHR044C | DOG1 | 2-deoxyglucose-6-phosphate phosphatase, similar to Dog2p, member of a family of low molecular weight phosphatases; confers 2-deoxyglucose resistance when overexpressed, in vivo substrate has not yet been identified. | + | - | - |
| YHR043C | DOG2 | 2-deoxyglucose-6-phosphate phosphatase, member of a family of low molecular weight phosphatases, similar to Dog1p, induced by oxidative and osmotic stress, confers 2-deoxyglucose resistance when overexpressed. | + | - | - |
| **Arabinono-1,4-lactone biosynthesis** | | | | | |
| YML086C | ALO1 | D-Arabinono-1,4-lactone oxidase, catalyzes the final step in biosynthesis of dehydro-D-arabinono-1,4-lactone, which is protective against oxidative stress. | + | - | - |
| YMR041C | ARA2 | NAD-dependent arabinose dehydrogenase, involved in biosynthesis of dehydro-D-arabinono-1,4-lactone; similar to plant L-galactose dehydrogenase. | + | - | - |
| **Proteins of unknown function** | | | | | |
| YDL144C | YDL144C | Putative protein of unknown function; green fluorescent protein (GFP)-fusion protein localizes to the cytoplasm and nucleus; YDL144C is not an essential gene. | + | - | - |
| YDR132C | YDR132C | Putative protein of unknown function. | + | - | - |
| YIL067C | YIL067C | Uncharacterized protein of unknown function. | + | - | - |
| YJR124C | YJR124C | Putative protein of unknown function; expression induced under calcium shortage. | + | - | - |
| YLR108C | YLR108C | Protein of unknown function; green fluorescent protein (GFP)-fusion protein localizes to the nucleus; YLR108C is not an esssential gene. | + | - | - |
| YOL137W | BSC6 | Protein of unknown function containing 8 putative transmembrane seqments; ORF exhibits genomic organization compatible with a translational readthrough-dependent mode of expression. | + | - | - |
| YPR127W | YPR127W | Protein of unknown function, differentially expressed during alcoholic fermentation; expression activated by transcription factor YRM1/YOR172W; green fluorescent protein (GFP)-fusion protein localizes to both the cytoplasm and the nucleus. | + | - | - |
| YPL103C | FMP30 | Protein of unknown function proposed to be involved in N-acylethanolamine metabolism; related to human NAPE-selective phospholipase D enzyme; native protein is detected in highly purified mitochondria in high-throughput studies. | + | - | - |
| YPL277C | YPL277C | Putative protein of unknown function; localized to the membranes; gene expression regulated by copper levels | + | - | - |
| YPR022C | YPR022C | Putative protein of unknown function; green fluorescent protein (GFP)-fusion protein localizes to both the cytoplasm and the nucleus and is induced in response to the DNA-damaging agent MMS. | + | - | - |
| **Chaperones** |  |  |  |  |  |
| YBR227C | MCX1 | Mitochondrial matrix protein; putative ATP-binding chaperone with non-proteolytic function; similar to bacterial ClpX proteins. | + | - | - |
| YMR038C | CCS1 | Copper chaperone for superoxide dismutase Sod1p, involved in oxidative stress protection; Met-X-Cys-X2-Cys motif within the N-terminal portion is involved in insertion of copper into Sod1p under conditions of copper deprivation. | + | - | - |
| **Nitrate metabolism** | | | | | |
| XP_752655 |  | nitrate transporter CrnA [Aspergillus fumigatus Af293]. | - | - | - |
| CAD28426 |  | nitrite reductase [Aspergillus fumigatus] (NiiA). | - | - | - |
| AAL85636 |  | nitrate reductase NiaD [Aspergillus fumigatus]. | - | 4.00E-22 | 5.00E-37 |
| **Proteases/peptidases** | | | | | |
| YBR286W | APE3 | Vacuolar aminopeptidase Y, processed to mature form by Prb1p. | + | - | - |
| YHR132C | ECM14 | Putative metalloprotease with similarity to the zinc carboxypeptidase family, required for normal cell wall assembly. | + | - | - |
| YIL108W | YIL108W | Putative metalloprotease. | + | - | - |
| **Aromatic amino acid metabolism** | | | | | |
| YGL202W | ARO8 | Aromatic aminotransferase I, expression is regulated by general control of amino acid biosynthesis. | + | - | - |
| YHR137W | ARO9 | Aromatic aminotransferase II, catalyzes the first step of tryptophan, phenylalanine, and tyrosine catabolism. | + | - | - |
| YER152C | YER152C | Protein with 2-aminoadipate transaminase activity; shares amino acid similarity with the aminotransferases Aro8p and Aro9p; YER152C is not an essential gene. | + | - | - |
| **Channels/transporters** | | | | | |
| YJL093C | TOK1 | Outward-rectifier potassium channel of the plasma membrane with two pore domains in tandem, each of which forms a functional channel permeable to potassium; carboxy tail functions to prevent inner gate closures; target of K1 toxin. | + | - | - |
| YBR296C | PHO89 | Na+/Pi cotransporter, active in early growth phase; similar to phosphate transporters of Neurospora crassa; transcription regulated by inorganic phosphate concentrations and Pho4p. | + | - | - |
| YIL023C | YKE4 | Zinc transporter; localizes to the ER; null mutant is sensitive to calcofluor white, leads to zinc accumulation in cytosol; ortholog of the mouse KE4 and member of the ZIP (ZRT, IRT-like Protein) family. | + | - | - |
| YKL221W | MCH2 | Protein with similarity to mammalian monocarboxylate permeases, which are involved in transport of monocarboxylic acids across the plasma membrane; mutant is not deficient in monocarboxylate transport. | + | - | - |
| YOL162W | YOL162W | V from 10118-10765, Uncharacterized ORF, "Putative protein of unknown function; member of the Dal5p subfamily of the major facilitator family". | + | - | - |
| **Repeat-induced point mutation (RIP)** | | | | | |
| gi\|154296783 | ref\|XP_001548821.1 | hypothetical protein BC1G_12419 [Botryotinia fuckeliana B05.10]. | - | - | - |
| gi\|154322765 | ref\|XP_001560697.1 | hypothetical protein BC1G_00725 [Botryotinia fuckeliana B05.10]. | - | - | - |
| gi\|2906004 | gb\|AAC03766.1 | C5-DN A-methyltransferase; Masc2 [Ascobolus immersus]. | - | - | - |
| **Mating type/cell cycle/budding** | | | | | |
| YBR276C | PPS1 | Protein phosphatase with specificity for serine, threonine, and tyrosine residues; has a role in the DNA synthesis phase of the cell cycle. | + | - | 9.00E-39 |
| YGL056C | SDS23 | One of two S. cerevisiae homologs (Sds23p and Sds24p) of the S. pombe Sds23 protein, which is implicated in APC/cyclosome regulation; involved in cell separation during budding. | + | - | - |
| YBR214W | SDS24 | One of two S. cerevisiae homologs (Sds23p and Sds24p) of the S. pombe Sds23 protein, which is implicated in APC/cyclosome regulation; involved in cell separation during budding; may play an indirect role in fluid-phase endocytosis. | + | - | - |
| YIL140W | AXL2 | Integral plasma membrane protein required for axial budding in haploid cells, localizes to the incipient bud site and bud neck; glycosylated by Pmt4p; potential Cdc28p substrate. | + | - | - |
| **ER quality control** | | | | | |
| YPL096W | PNG1 | Conserved peptide N-glycanase required for deglycosylation of misfolded glycoproteins during proteasome-dependent degradation, localizes to the cytoplasm and nucleus, interacts with the DNA repair protein Rad23p. | + | - | - |
| YHR176W | FMO1 | Flavin-containing monooxygenase, localized to the cytoplasmic face of the ER membrane; catalyzes oxidation of biological thiols to maintain the ER redox buffer ratio for correct folding of disulfide-bonded proteins. | + | - | - |
| YBR015C | MNN2 | I from 269503-267710, reverse complement, Verified ORF, "Alpha-1,2-mannosyltransferase, responsible for addition of the first alpha-1,2-linked mannose to form the branches on the mannan backbone of oligosaccharides, localizes to an early Golgi compartment". | + | - | - |
| YJL186W | MNN5 | Alpha-1,2-mannosyltransferase, responsible for addition of the second alpha-1,2-linked mannose of the branches on the mannan backbone of oligosaccharides, localizes to an early Golgi compartment. | + | - | - |
| **Others** |  |  |  |  |  |
| YLL057C | JLP1 | Fe(II)-dependent sulfonate/alpha-ketoglutarate dioxygenase, involved in sulfonate catabolism for use as a sulfur source; contains sequence that resembles a J domain (typified by the E. coli DnaJ protein); induced by sulphur starvation. | + | - | - |
| YDR465C | RMT2 | Arginine methyltransferase; ribosomal protein L12 is a substrate. | + | - | - |
| YNL229C | URE2 | Nitrogen catabolite repression transcriptional regulator that acts by inhibition of GLN3 transcription in good nitrogen source; has glutathione peroxidase activity and can mutate to acquire GST activity; altered form creates [URE3] prion | + | - | - |
| YOR388C | FDH1 | NAD(+)-dependent formate dehydrogenase, may protect cells from exogenous formate. | + | 3.00E-14 | 1.00E-22 |
| YDR242W | AMD2 | Putative amidase. | + | - | - |
| YMR302C | YME2 | Integral inner mitochondrial membrane protein with a role in maintaining mitochondrial nucleoid structure and number; mutants exhibit an increased rate of mitochondrial DNA escape; shows some sequence similarity to exonucleases | + | - | - |
| YJL145W | SFH5 | Non-classical phosphatidylinositol transfer protein (PITP); exhibits PI- but not PC-transfer activity; localizes to the peripheral endoplasmic reticulum, cytosol and microsomes; similar to Sec14p | + | - | - |
| YLR047C | FRE8 | Protein with sequence similarity to iron/copper reductases, involved in iron homeostasis; deletion mutant has iron deficiency/accumulation growth defects; expression increased in the absence of copper-responsive transcription factor Mac1p. | + | - | - |
| YLR278C | YLR278C | Zinc-cluster protein; GFP-fusion protein localizes to the nucleus; mutant shows moderate growth defect on caffeine; has a prion-domain like fragment that increases frequency of [URE3]; YLR278C is not an essential gene. | + | - | - |
| YIL162W | SUC2 | Invertase, sucrose hydrolyzing enzyme; a secreted, glycosylated form is regulated by glucose repression, and an intracellular, nonglycosylated enzyme is produced constitutively. | + | - | - |
| YDR030C | RAD28 | Protein involved in DNA repair, related to the human CSA protein that is involved in transcription-coupled repair nucleotide excision repair. | + | - | - |
