# Supplementary material for: De novo Analysis of the Epiphytic Transcriptome of the Cucurbit Powdery Mildew Fungus Podosphaera xanthii and Identification of Candidate Secreted Effector Proteins
Source: PLoS One. 2016 Oct 6;11(10):e0163379. doi: 10.1371/journal.pone.0163379 (PMC5053433; doi:10.1371/journal.pone.0163379)
Supplement: S4 Table — Categories “Pathogenesis” and “Host interaction”, and the list of CSEPs are shown in Tables 2 and 3, respectively. (DOCX) [file pone.0163379.s006.docx]

**S4 Table. Annotation of the *P. xanthii* epiphytic secretome (rest of categories).** Categories “Pathogenesis” and “Host interaction”, and the list of CSEPs are shown in Table 2 and Table 3, respectively.

| **Sequence ID** | **Description** | | **E-value^a^** | **Sequence identity (%)** | **Subject ID^b^** | **Protein length (aa)** | |
| --- | --- | --- | --- | --- | --- | --- | --- |
| **Cell wall modification** | | |  |  |  |  | |
| Contig1986 | 1,3-beta-glucanosyltransferase | | 1.00E-178 | 73% (311/424) | P0C7S9 | 448 | |
| Contig3617 | Beta-glucanosyltransferase gel2 | | 1.00E-138 | 55% (237/424) | P0C954 | 497 | |
| Contig5273 | carboxylesterase | | 1.00E-105 | 48% (157/321) | P79066 | 364 | |
| Contig4643 | carboxylesterase family protein | | 4.00E-34 | 33% (109/330) | P17573 | 416 | |
| Contig2746 | Carboxylesterase | | 1.00E-116 | 43% (168/387) | A7E504 | 577 | |
| Contig2727 | Carboxylesterase | | 1.00E-113 | 46% (227/490) | A7E504 | 576 | |
| Contig1889 | Carboxypeptidase S1 | | 1.00E-174 | 62% (206/332) | A1C5M4 | 478 | |
| Contig5329 | Carboxypeptidase S1 | | 1.00E-176 | 62% (294/471) | A1C5M4 | 495 | |
| Contig_ c813_mira | Carboxypeptidase S1 | | 1.00E-180 | 65% (295/452) | A1C5M4 | 476 | |
| Contig3302 | Carboxypeptidase Y | | 0 | 69% (372/537) | A7F4H5 | 530 | |
| Contig4501 | Cell wall glucanase (Utr2) | | 1.00E-129 | 63% (219/347) | Q4WI46 | 420 | |
| Contig7612 | Cell wall glucanase | | 1.00E-73 | 44% (161/362) | A7EGQ5 | 376 | |
| Contig3504 | Cell wall glucanase | | 5.00E-76 | 46% (159/345) | Q8J0P4 | 460 | |
| Contig3744 | Cell wall protein | | 4.00E-12 | 28% (54/188) | G2Q028 | 289 | |
| Contig2787 | Chitin deacetylase | | 1.00E-114 | 63% (199/313) | Q96VN0 | 325 | |
| Contig6385 | Chitin deacetylase | | 1.00E-109 | 70% (115/162) | Q96VN0 | 318 | |
| Contig4394 | Chitinase A | | 1.00E-140 | 59% (231/388) | P48827 | 447 | |
| Contig6254 | Cuticle-degrading serine protease | | 1.00E-54 | 38% (144/376) | G1X8P8 | 402 | |
| Contig_c42_mira | Cuticle-degrading serine protease | | 5.00E-55 | 38% (146/376) | G1X8P8 | 402 | |
| Contig2285 | endoglucanase | | 2.00E-76 | 37% (149/395) | Q96WQ9 | 381 | |
| Contig_c153_mira | endoglucanase | | 2.00E-76 | 37% (149/395) | Q96WQ9 | 381 | |
| Contig4553 | Extracellular chitinase | | 1.00E-155 | 60% (229/380) | Q96VN1 | 484 | |
| Contig2760 | Glucan 1,3-beta-glucosidase | | 0 | 78% (334/423) | Q96V64 | 425 | |
| Contig_c19040_mira | Glucan 1,3-beta-glucosidase | | 2.00E-75 | 57% (89/154) | H1V3J7 | 324 | |
| Contig2735 | Glucan 1,3-beta-glucosidase | | 1.00E-138 | 58% (235/405) | G2XSV1 | 419 | |
| Contig3779 | Glucan 1,3-beta-glucosidase | | 1.00E-109 | 67% (191/282) | P15703 | 303 | |
| Contig3143 | Glycoside hydrolase, family 76 | | 1.00E-134 | 46% (262/565) | A7F2F9 | 560 | |
| Contig6729 | Glycosyl hydrolase family 16 | | 1.00E-110 | 59% (198/334) | P53301 | 374 | |
| Contig7624 | Polysaccharide deacetylase | | 1.00E-104 | 53% (188/349) | Q06702 | 355 | |
| Contig4157 | polysaccharide deacetylase | | 8.00E-98 | 46% (193/414) | G2YBV8 | 772 | |
| Contig2716 | Herpes_gp2 multi-domain protein | | 4.00E-06 | 24% (30/122) | G2YMX6 | 463 | |
| Contig_c4665_mira | Similar to beta-1,6-glucan boisynthesis protein (Knh1) | | 6.00E-45 | 41% (106/253) | E4ZHV8 | 259 | |
| **Reproduction** |  | |  |  |  |  | |
| Contig3014 | Bc-hch | | 0 | 61% (484/783) | A7E572 | 782 | |
| Contig3643 | Nima-interacting protein | | 0 | 68% (285/414) | H0EZ47 | 427 | |
| **Cell wall organization** | |  |  |  |  |  | |
| 3600_euler | GPI anchored serine-threonine rich protein | | 2.00E-19 | 43% (51/116) | F0XHH6 | 263 | |
| Contig_c375_mira | GPI anchored serine-threonine rich protein | | 2.00E-19 | 43% (51/116) | F0XHH6 | 324 | |
| Contig2851 | GPI-anchored cell wall organization protein ecm33 | | 3.00E-63 | 37% (149/402) | Q4WNS8 | 394 | |
| Contig3656 | Fasciclin domain-containing protein | | 5.00E-38 | 32% (100/308) | B8M437 | 333 | |
| **Transport** |  | |  |  |  |  | |
| Contig3113 | Endosomal P24B protein | | 2.00E-83 | 73% (145/198) | P32803 | 202 | |
| **Nuclease activity** |  | |  |  |  |  | |
| Contig1341 | extracellular putative DNase | | 3.00E-28 | 44% (76/171) | G2Y3K8 | 167 | |
| **Lipid metabolism** |  | |  |  |  |  | |
| Contig3187 | Herpes_gp2 domain containing protein | | 7.00E-06 | 27% (37/137) | A7EZK6 | 460 | |
| Contig2916 | COesterase multi-domain protein | | 6.00E-80 | 27% (152/553) | D2CQL4 | 573 | |
| Contig3111 | autophagy related lipase Atg15 | | 1.00E-137 | 47% (247/524) | Q0CXU6 | 568 | |
| **Carbohydrate metabolism** | | |  |  |  |  | |
| Contig2963 | Glycoside hydrolase family 16 protein | | 5.00E-72 | 44% (158/358) | G2Y6Q9 | 384 | |
| **Protein catabolism** |  | |  |  |  |  | |
| Contig3224 | Tripeptidyl-peptidase sed3 | | 5.00E-37 | 37% (50/135) | Q70GH4 | 606 | |
| Contig1446 | Serine carboxypeptidase (CpdS) | | 1.00E-172 | 57% (305/529) | P52719 | 547 | |
| Contig5914 | Serine protease | | 1.00E-173 | 65% (302/460) | C5P4Z8 | 464 | |
| Contig_c3_mira | Serine protease | | 1.00E-173 | 66% (304/460) | C5P4Z8 | 464 | |
| Contig3173 | Aspartic proteinase | | 6.00E-73 | 39% (164/414) | Q4WNV0 | 499 | |
| Contig2714 | Aspartyl protease | | 1.00E-131 | 58% (269/463) | Q4WZS3 | 483 | |
| **Cellular homeostasis** | |  |  |  |  |  | |
| Contig2825 | Mannosyl-oligosaccharide alpha-1,2-mannosidase 1B | | 1.00E-169 | 58% (300/512) | Q12563 | 524 | |
| Contig2878 | Function: B. circulans aman6 is a yeast mannan backbone degrading enzyme | | 1.00E-94 | 50% (182/359) | G2YAT2 | 385 | |
| Contig8386 | glutaminase GtaA | | 0 | 60% (416/686) | Q9UVX9 | 691 | |
| **Response to stress** |  | |  |  |  |  | |
| Contig6436 | stress response protein (Ish1) | | 1.00E-140 | 46% (264/567) | Q03104 | 568 | |
| **Regulation of biological process** | | |  |  |  |  | |
| Contig2798 | Superoxide dismutase [Cu-Zn] | | 5.00E-67 | 53% (137/255) | G2Y619 | 247 | |
| Contig2733 | Tyrosinase | | 1.00E-83 | 33% (201/595) | A7BHQ9 | 584 | |
| **Carbohydrate metabolism** | | |  |  |  |  | |
| Contig5877 | WSC domain containing protein | | 4.00E-38 | 36% (92/253) | G2Y4G7 | 273 | |
| Contig_c192_mira | WSC domain containing protein | | 8.00E-29 | 32% (76/235) | G2Y4G7 | 261 | |
| **Protein metabolism** |  | |  |  |  |  | |
| Contig8906 | LisH domain-containing protein | | 2.00E-07 | 26% (37/141) | Q9P785 | 353 | |
| **Riboflavin biosynthesis** | | |  |  |  |  | |
| Contig8415 | 6,7-dimethyl-8-ribityllumazine synthase | | 1.00E-34 | 65% (80/123) | Q9UVT8 | 163 | |
| **Signal transduction** | | |  |  |  |  | |
| Contig_c15685_mira | SH3 and Ded_cyto domain protein | | 2.00E-54 | 48% (130/267) | G2YD58 | 238 | |
| ^a^E-values were obtained after Blast analysis | | | | | | |  |
| ^b^GenBank accession number | | | | | | |  |
